# Dr. F. F. G. Eggert

**Published:** 1838-01

**Authors:** 


### DR. F. F. G. EGGERT.

Franz Friedrich Gottlob Eggert was born at Eisleben, on the 15th August, 1778. He studied medicine, first at Jena, and afterwards at Leipzig, where he graduated in 1802. From this time till 1816, he practised at Querfurth; and from 1816 until his death, which took place on the 23d August, 1836, in his native town of Eisleben. Besides many practical and critical articles in the medical journals, Dr. Eggert was author of the following works: 1. Ueber die Wassersucht. Leipzig, 1817. 2. Ueber das Wesen und die Heilung des Croups. Hannover, 1820.—3. Die Organische Natur des Menschen. 2 Bde. Leipzig, 1828.—4. Der gewaltsame Tod ohne Verletzung. Berlin, 1832.

